# How Does City Size Affect the Cost of Household Travel? Evidence from an Urban Household Survey in China

**DOI:** 10.3390/ijerph19116890

**Published:** 2022-06-04

**Authors:** Zhentao Li, Tianzi Li

**Affiliations:** 1 Northeast Asian Studies College, Jilin University, Changchun 130012, China; ztli18@mails.jlu.edu.cn; 2 Northeast Asian Research Center, Jilin University, Changchun 130012, China

**Keywords:** city size, travel cost, spatial distance, traffic congestion, public transportation, urban road

## Abstract

Travel costs are critical to the sustainable development of cities. This paper used Urban Household Survey (UHS) data from 2002 to 2014 and constructed a comprehensive city-size index from the perspectives of population and urban space to empirically test the impact of city size on the cost of household travel. The main results are as follows: (1) There is a significant positive correlation between city size and the cost of household travel. The internal mechanism is that city size affects the cost of household travel by increasing spatial distance and traffic congestion. (2) Increasing public transportation and per capita road area can restrain the positive impact of city size on the cost of household travel; moreover, the restraining effect of public transportation is stronger than that of per capita road area. (3) The impact of city size on the cost of household travel for sub-provincial cities is smaller than that for ordinary prefecture-level cities; in addition, there is an inverted U-shaped relationship between city size and the cost of household travel. This paper deepens the understanding of the impact of city size on travel costs, providing research support for the healthy development of cities in China.

## 1. Introduction

Over the past four decades, China has undergone tremendous urbanization, resulting in a dramatic increase in the numbers and sizes of cities. From 1978 to 2020, the number of cities increased from 193 to 687, and the urbanization rate increased from 17.9% to 63.89%, with an average annual growth rate of 1.09% [1]. Although the expansion of city sizes has enhanced the efficiency of urban resource allocation and increased residents’ incomes [2,3,4], the expansion of city sizes has also brought a series of problems, including air pollution, loss of arable land and increased spending on public services [5,6,7]. In addition to these negative consequences, there is a particularly worrying economic impact: increased travel costs. The increase in travel costs has seriously affected the daily travel behavior of urban residents. Therefore, it is necessary to explore the internal mechanism of the impact of city size on travel costs. This is of great significance for improving the welfare of urban residents, sustainable urban development and related policy formulation.

City size has long been considered an important factor affecting travel costs. However, its effects are theoretically uncertain, and empirical studies are inconclusive. Several studies support the positive relationship between city size and travel costs [8,9,10,11]. The main reason is that commuting distances increase as cities grow. People’s choices of workplaces are limited not by what is available nearby but by what is available in the whole city [10]. Coevering and Schwanen conducted a multiple regression analysis of 31 cities in Europe, Canada and the United States. They found a significant positive correlation between travel distance and population size [12]. Melo, Graham and Noland found that commute distances in England and Wales tended to increase with urban size [13]. On the other hand, some scholars argue that the expansion of city size will reduce travel costs. This relationship is affected by the internal spatial structure of the city [14]. People have more opportunities to live closer to their workplaces in the polycentric city model. This phenomenon is called co-location, and it reduces commuting distances [11,13]. Some studies found that polycentric structures result in lower average distances than monocentric structures [15,16]. For example, using data from a household interview survey, Zhao found that polycentric development can reduce the number of suburb-to-center commutes and overall commuting distances [17]. Finally, a third branch of the literature shows that the nexus between city size and travel costs is uncertain. Gordon (1989) found no clear relationship between city size and trip length, time or speed in data from the National Personal Transportation Research Survey in 1977 and 1983–1984 [18].

Based on the literature, it is clear that the nexus between city size and travel costs has been extensively explored in western countries with a long history of industrial and infrastructure development. However, the research on the relationship between city size and travel costs in China started relatively late. Sun was first to use the macro data from 164 cities in China to empirically study the relationship between city size and commuting costs. He found a positive correlation between commuting time and the size of the urban population [19]. Subsequently, Engelfriet and Koomen used Baidu commuting data to select 30 large Chinese cities as research objects. The study found that with a population increase of 1%, commuting distance increases by 31 m and commuting time by 6 s [11]. Rao used the commuting data released in the *2018 China Urban Transportation Report* to select the top 100 cities with traffic congestion in China as research objects. The study found that city size was significantly negatively correlated with urban commuting efficiency. The larger the urban built-up area, the longer commute time and commute distance [20].

Our review of the literature reveals limitations in the existing research. First, most empirical studies on travel costs have focused on commuting travel or personal travel. However, this may not be the case in practice. In fact, people are very likely to make a series of trips (chained trips) between their origin and their final destination. In addition, some travel behaviors may be influenced by the joint decision-making of family members. Therefore, the impact of city size on travel costs cannot be accurately assessed from individual travel or single-trip travel. Second, city size includes the sizes of the urban population and the urban built-up area. Although some studies have compared the impacts of different city sizes on travel costs, most of these studies are based on a single urban population size or urban built-up area. However, the travel cost is the result of the combined effect of urban population size and urban built-up area, so there is a lack of comprehensive city size indicators to analyze the impact on travel cost. Third, the study of city size on travel costs from micro-perspectives is mainly concentrated in western countries. After taking into account individual micro-characteristics and temporal trends, does the impact on travel costs differ across cities as city size increases for Chinese cities? In addition, is the relationship with travel costs linear?

The main contributions of this paper are as follows. First, different from the existing research on the impact of city size on travel costs in China, this paper constructs a comprehensive city size index from the micro-perspective of households to explore the impact of city size on household travel costs and alleviates the endogeneity problem through instrumental variables. Second, based on the existing research, this paper proposes a theoretical framework to analyze how city size affects and reduces the cost of household travel, and it uses an econometric model to test the internal mechanism of the impact of city size on travel costs. Third, from the perspective of urban heterogeneity, this paper analyzes the differential effect of the impact of city size on the cost of household travel. There is a nonlinear relationship between the impact of city size on the cost of household travel, providing a realistic basis for reducing the cost of household travel.

The rest of this paper is organized as follows: Section 2 proposes the hypotheses about city size and the cost of household travel. Section 3 presents the research methodology and data sources. Section 4 uses the data from the Urban Household Survey (UHS) to empirically analyze the impact of city size on the cost of household travel and to test its theoretical mechanism. Section 5 is about the conclusions, recommendations and discussions of future research.

## 2. Research Hypotheses

**Figure 1:**
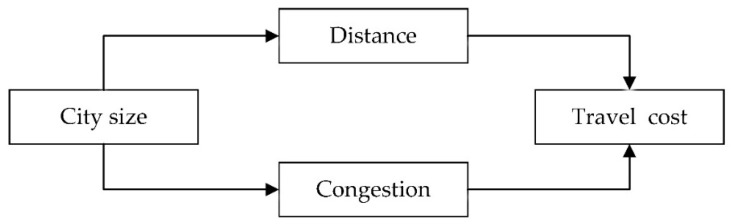
The influence pathways of city size on travel costs.

In the standard city model, the cost of household travel depends on distance [21]. City size is an important factor affecting travel distance. In particular, most travel destinations are outside the residential areas. The travel distance of residents may be more affected by the spatial distance between their dwellings and the potential travel destinations [22]. In addition, the city size determines the scope of urban residents’ activities [20]. Therefore, the spatial distance to the potential travel destination increases with the city size, resulting in an increase in the travel cost for urban residents. On the contrary, some studies have shown that with the increase in city size, the urban spatial structure shifts from monocentric cities to polycentric cities, shortening the average travel distance. This is because people have more opportunities to live closer to their workplaces and achieve work-housing balance in a polycentric city model, thereby reducing travel costs [13]. However, there may be an increase in non-work travel in polycentric urban areas because of the increases in family car ownership, availability of time and demand for goods and services [23]. In addition, most cities in China are dominated by a single center [24], and the travel types of urban residents are mainly centripetal travel and arbitrary direction travel [25]. The influence pathways of city size on travel cost are shown in Figure 1, and we propose the following hypotheses:

**Hypothesis** **1** **(H1).** *City size increases the cost of household travel.*

**Hypothesis** **2** **(H2).** *City size can affect the cost of household travel by increasing the spatial distances.*

The increase in city size will bring some agglomeration diseconomies. This is usually explained by urban problems such as traffic congestion [26]. When the number of vehicles on the road increases to a certain extent, the distance between vehicles will decrease, and the speed of vehicles will naturally slow down or even stop, resulting in the traffic congestion [27]. With the expansion of city size, the total traffic flow of urban residents will show a structural increase. However, the proportion of urban road area has not changed structurally, so traffic congestion will inevitably become more serious. According to Downs, the most important cause of traffic congestion is the growth of city size. Almost all large urban areas in the world will experience severe traffic congestion because of the faster population growth in urban areas [28]. Stopher also came to a similar conclusion that an increase in the size of the urban population will lead to an increase in the degree of congestion [29]. Due to the existence of traffic congestion, the extra fuel consumption of private cars and the increase in taxi service fees lead to higher travel costs. Accordingly, we propose the following hypothesis:

**Hypothesis** **3** **(H3).** *City size can affect the cost of household travel by increasing congestion.*

Urban roads are the key link connecting the inner areas of a city. They determine the smoothness of material and information exchanges between the inner areas of the city. The expansion of city size is not only the agglomeration of the population to the city but also the expanding area of the urban land. The urban development model in China is to spread along the edge of the original urban built-up area to the periphery, forming fragmented land use types [30,31]. In addition, the third industry gathers in the city center, forming a new employment center [32]. However, in the context of rapid urbanization, urban road construction lags urban development, and the spatial distribution of urban roads is uneven [30]. Therefore, the road mileage increased and road width was expanded in the process of increasing the city sizes. In particular, it is necessary to build a ring line connecting the edges of the city and a fast passage connecting the urban central area and the edges. First, the ring line can reduce detours and shorten the travel distance of residents; second, it can alleviate urban road congestion and improve travel efficiency. Therefore, we propose the following:

**Hypothesis** **4** **(H4).** *Increasing the per capita area of paved roads can restrain the positive impact of city size on the cost of household travel.*

The choice of travel mode is usually regarded as an application of consumer choice theory. People maximize their personal utility based on making rational choices from among competing alternatives [33,34]. When deciding how to get from point A to point B, urban residents will weigh the comparative travel times, costs and other attributes [35]. The choices of motorized travel modes for urban residents are mainly divided into public transportation and personal transportation. Compared with personal transportation, urban public transportation has the advantages of low occupancy rate, high passenger load rate and low price. With the expansion of city sizes, the development of urban public transportation and the improvement of the accessibility of public transportation can reduce the dependence on cars and encourage travelers to switch to public transportation for travel [36]. Therefore, the cost of household travel can be reduced. Wall and McDonald showed that the number of bus passengers significantly increased with the increase in the frequency of bus services in Winchester, UK [37]. Redman, Friman and Gärling reached the same conclusion [38]. A survey of car users reducing car use during commuting found that public transportation with higher service frequency is more attractive to car users [39]. Therefore, we propose the following:

**Hypothesis** **5** **(H5).** *Increasing the number of public transportation vehicles can restrain the positive impact of city size on the cost of household travel.*

## 3. Research Methods and Data Sources

### 3.1. Model

This paper mainly studied the impact of city size on the cost of household travel. The following model was constructed:

(1) $$\ln\;\left({\mathrm{travel}\;\cos t}_{\mathrm{ijt}}\right)=\beta_{0}+\beta_{1}{\mathrm{size}}_{\mathrm{jt}}+\beta_{2}\mathrm{CV}+{\;\beta }_{3}D+y_{t}+\varepsilon$$

where the subscript i represents the household, j represents the city and t represents the time. The variable ${\mathrm{travel}\;\cos t}_{\mathrm{ijt}}$ represents the cost of household travel, and ${\;\mathrm{size}}_{\mathrm{jt}}$ is the core explanatory variable, representing the city size. CV is the control variable that can reduce the estimation error caused by the omitted variable (the choice of the control variable will be explained in detail later); D represents a series of urban characteristic variables that are independent of time (including river, terrain, disbeijing, disshanghai, longitude, and latitude, etc.); $y_{t}$ is the time fixed effect and ε is the random error.

### 3.2. Variables Selection

#### 3.2.1. Explained Variables

This paper selects the cost of household travel (travel cost) as the explained variable; the unit is yuan per household. The UHS records the detailed travel expenses of each household in the city in one year. The cost of household travel includes the cost of purchasing car, fuel and transportation services and the cost of public transport and taxis in the city.

#### 3.2.2. Explanatory Variable

Urbanization rate refers to the proportion of urban population to total population (including agricultural and non-agricultural). City size refers to the size of a city expressed in terms of the urban population and the urban built-up area. This paper constructs a comprehensive city size index (${\mathrm{size}}_{\mathrm{it}}$) from the urban population (pop) and the urban built-up area (area) indicators. Because a single indicator cannot accurately measure the city size, we constructed a comprehensive city size indicator from the perspective of urban population and built-up area based on Zhou’s work [40]. Weight refers to the importance of a factor or indicator relative to something. Many studies have shown that the weights of all factors or indicators add up to 1 [40,41]. Because the urban population affects the travel flow, the urban built-up area affects the travel scope, and the travel cost is affected by the urban built-up area and the population. Based on Liu’s work [42], we assumed that the urban population is as important as the built-up area. Therefore, we set the weight to 0.5. The index construction process is as follows:

(2) $${\mathrm{size}}_{\mathrm{it}}=\left(0.5\times \frac{{\mathrm{pop}}_{\mathrm{ij}}-\min\left({\mathrm{pop}}_{\mathrm{ij}}\right)}{\max\left({\mathrm{pop}}_{\mathrm{ij}}\right)-\min\left({\mathrm{pop}}_{\mathrm{ij}}\right)}+0.5\times \frac{{\mathrm{area}}_{\mathrm{ij}}-\min\left({\mathrm{area}}_{\mathrm{ij}}\right)}{\max\left({\mathrm{area}}_{\mathrm{ij}}\right)-\min\left({\mathrm{area}}_{\mathrm{ij}}\right)}\right)$$

where ${\mathrm{pop}}_{\mathrm{ij}}$ represents the urban population. The unit of population is 10,000; $\min\;\left({\mathrm{pop}}_{\mathrm{ij}}\right)$ represents the minimum value of the urban population; $\max\;\left({\mathrm{pop}}_{\mathrm{ij}}\right)$ represents the maximum value of the urban population; ${\mathrm{area}}_{\mathrm{ij}}$ represents the urban built-up area; the unit of area is square kilometers; $\min\;\left({\mathrm{area}}_{\mathrm{ij}}\right)$ represents the minimum value of the urban built-up area; and $\max\;\left({\mathrm{area}}_{\mathrm{ij}}\right)$ represents the maximum value of the urban built-up area. This paper used nighttime light data to extract the urban built-up area [43,44,45]. The geographic information of the urban space extracted by the night light time data is more detailed than the statistical data. Scholars mostly set the urban brightness threshold based on their own experience. Harari argued that areas with a light threshold of more than 35 can be regarded as urban areas [46]. However, the economic development level of China is higher than that of India. Areas with light brightness values greater than 40 may be urban areas. Therefore, the light threshold in this paper is 40.

#### 3.2.3. Mediating Variables

Congestion (congestion): We used the number of civilian cars in the city to show the urban congestion. The more cars a city has, the more likely they are to cause traffic congestion [29]. At the same time, it is difficult to obtain historical data on urban traffic congestion. Therefore, we used the number of civilian cars as a proxy variable for traffic congestion. Congestion is expressed in the logarithm form.

Distance (distance): The spatial distance. The average value of the linear distance between all grids in the city can reflect the urban spatial distance. First, existing research shows that city size determines the range of activities of residents. The travel types of urban residents are mainly centripetal travel and arbitrary direction travel [25]. Second, it is difficult to obtain travel distance data for each resident. Therefore, the travel distance of residents is replaced by the average straight-line distance between the grids [47]. The calculation method is as follows: If there are N grids in a city, the number of distances between grids is [N × (N − 1)]. Then, the average value will be calculated. The unit is km expressed in logarithm form.

#### 3.2.4. Moderator Variable

**Figure 2:**
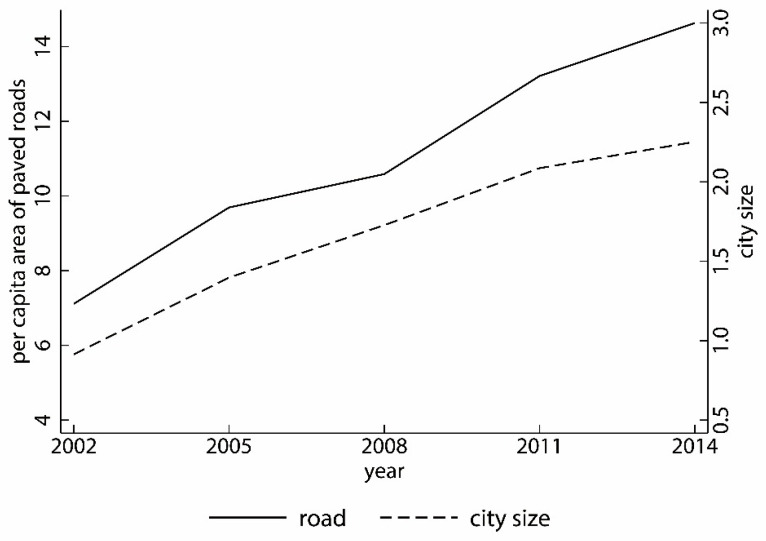
Trends in city size and per capita area of paved roads.

Road (road): The per capita area of paved roads in the city can reflect the level of urban infrastructure. The greater the per capita area of paved roads, the less congestion on urban roads. Therefore, this can reduce travel costs; road is expressed in logarithm form. Figure 2 shows the trends in city size and per capita area of paved roads.

**Figure 3:**
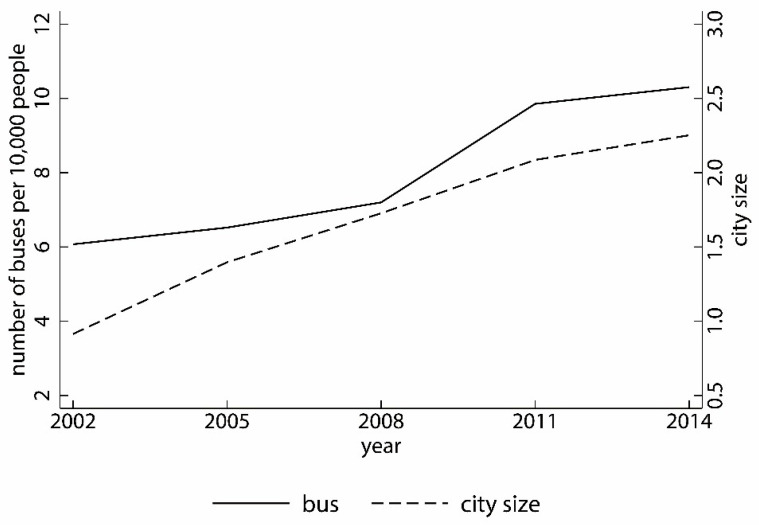
Trends in city size and the number of buses per 10,000 people.

Bus (bus): The number of public transportation vehicles per 10,000 people in the city. The greater the number of buses per 10,000 people, the greater the likelihood that residents will choose public transport for travel, thereby reducing the cost of household travel; bus is expressed in logarithm form. Figure 3 shows the trends in city size and the number of buses per 10,000 people.

#### 3.2.5. Control Variables

The control variables mainly include household socioeconomic characteristics, urban characteristics and physical geographic characteristics.

Household socioeconomic characteristics:

Age (age) is one of the commonly used socioeconomic factors. Several studies have shown that travel distance is inversely proportional to age [48,49,50]. This paper selects the average age of family members as the proxy variable for age.

Education (education): It is widely believed that the more educated people are, the more likely they are to seek employment in a wider area for a higher income [51,52]. This paper selects the average education level of household members as the proxy variable for education in this paper. We set no elementary school as 1, literacy class as 2, elementary school as 3, junior high school as 4, senior high school as 5, technical secondary school as 6, junior college as 7, university undergraduate as 8 and postgraduate and above as 9.

Minors (minors): Studies have found that families with minors will increase the frequency of private car use and the number of extra trips [53]. Family member of minors is a dummy variable. For the question of whether there are minors in the family, 1 means yes, and 0 means no.

Family size (family size): The total family population represents the family size. Studies have shown that the larger the family, the higher the transportation cost [12].

Economic sector (sector): The cost of travel is closely related to the economic sector. In China, the state-owned economic sector is relatively stable, the commuting distance is relatively fixed and the 8 h work system is strictly adhered to. Therefore, people have time to increase other travel activities. However, there is the possibility of changing jobs in the private sector, and the commuting distances are not fixed. Therefore, different economic sectors affect travel costs. For family members working in the state-owned economic sector, 1 means yes, and 0 means no.

Income is an important factor affecting travel costs. The higher the income, the more likely residents will spend more on travel [54]. Income is a set of variables, including wage income (wage), operational income (operational), property income (property) and transfer income (transfer), to examine the impacts of different income types on travel costs. The unit is yuan per household expressed in logarithm form.

Urban characteristics:

Density (density): Population density is one of the important factors affecting travel costs. It is generally believed that a higher urban population density will lead to a decrease in the proportion of car travel and an increase in the proportion of non-motorized travel, thereby reducing travel costs [55]. The unit is person/km^2^ in logarithm form.

Price (price): The price level. The transportation costs are high in areas with high prices. This paper used the minimum hourly wage to measure regional price levels because the local minimum wage is based on the local living expenses and price levels. The unit is yuan in logarithm form.

Government intervention (government): Represented by the ratio of government fiscal expenditure to GDP. Strong government intervention (such as a spatial plan) may promote job-housing balance, which in turn affects travel costs [56].

Air pollution (PM 2.5): This paper used a concentration of PM 2.5 to indicate the urban air quality. The higher the concentration of PM 2.5, the worse the urban air quality. PM 2.5 is more harmful to human health than PM 10 [57]. In addition, ozone is not easily detected, especially on hot sunny days when ozone can reach unhealthy levels [58]. As the concentration of PM 2.5 rise in urban areas, residents reduce unnecessary travel. The unit is micrograms/m^3^ in logarithm form.

Subway (subway): The number of subway lines in the city. The subway is fast and convenient with low cost. Therefore, the subway can reduce the cost of urban household travel; it is expressed in logarithm form.

Urban spatial form (spatial form): The largest patch area in the urban built-up area divided by the urban built-up area, used to reflect the urban spatial form. The larger the value, the more concentrated the urban land and the more compact the urban spatial form. Existing research shows that compact urban forms can reduce travel costs [59].

Physical geographic characteristics:

River (river): Rivers in a city is a dummy variable where 1 means yes there are rivers and 0 means no. Rivers divide an urban area into several parts. This reduces the accessibility of various areas of the city, leading to an increase in travel costs.

Terrain (terrain): The degree of undulation in the land of cities. Terrain undulations may affect the cost of household travel. This is because terrain undulations create fragmented land use patterns, thereby increasing the travel distances of residents. The calculation method for terrain undulations refers to Feng [60].

Temperature (temperature): The average annual temperature of the city. It is closely related to the travel of residents. As the temperature increases, the outdoor activities of urban residents will increase. The unit is °C in logarithm form.

Precipitation (precipitation): The average annual precipitation in the city. The regional precipitation may affect the travel of urban residents. This is because the more precipitation in the area, the fewer the travel activities of urban residents. The unit is mm/m^2^ in logarithm form.

We added a set of urban geographical feature variables that do not change over time based on the processing methods of Faberman and Freedman and Duranton and Turner [61,62]. This can solve the possible errors of missing variables. These variables include the latitude (latitude) and longitude (longitude) of the city government and the distance between each city and Beijing (disbeijing) and Shanghai (disshanghai). In addition, we also added province dummy variables and year dummy variables to the model. When controlling for the above variables, there may be the problem of missing variables. Therefore, we used instrumental variables for endogeneity testing. The statistics of the variables are listed in Table 1.

### 3.3. Data Sources

**Figure 4:**
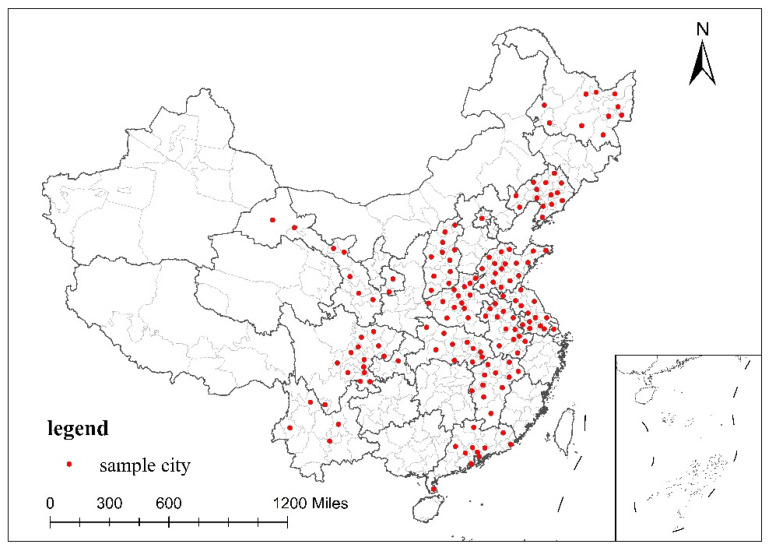
The spatial distribution of the sample cities.

The primary dataset used in this paper was from the UHS of the National Bureau of Statistics of China. This survey began in 1985. It took households in the municipal districts and townships of counties as the survey objects and showed the socioeconomic status of urban households. Due to data access restrictions, we used a part of the full UHS samples. It includes 153 cities in China from 2002, 2005, 2008, 2011 and 2014. These data were distributed across 16 provinces: Beijing, Shanxi, Liaoning, Heilongjiang, Shanghai, Jiangsu, Anhui, Jiangxi, Shandong, Henan, Hubei, Guangdong, Chongqing, Sichuan, Yunnan and Gansu. This is wide representation in terms of geographic location and economic development level. The 153 sample cities are shown in Figure 4. This paper focused on the cost of household travel in the municipal districts. Therefore, only the sample of households in the questionnaire in the municipal districts was retained in this paper; the sample of households in a county and missing and unreasonable data were eliminated. Finally, a total of 100,869 valid data points were obtained in this paper.

The night lighting data were from 34 reports issued by the National Geophysical Data Center of the National Oceanic and Atmospheric Administration of the United States during the 22 years from 1992 to 2013. EOG used pre-dawn data to extend the annual DMSP night lighting time series to 2019. The original night lighting data cannot be directly used to study the city size [63]. Therefore, we corrected the night lighting data from 1992 to 2019 by the method proposed by Liu and Li [45,64]. The processed night lighting data can better reflect the expansion of construction land in prefecture-level cities and are more suitable for cross-year and cross-regional comparisons.

In addition to the UHS’s data, this paper also used the National Bureau of Statistics *China Urban Statistical Yearbook*, *China Land and Resources Statistical Yearbook* and *China Urban Construction Statistical Yearbook* in the empirical analysis. The data on temperature and precipitation were from the China Meteorological Data Service Centre [65]. The PM 2.5 data were from the Atmospheric Composition Analysis Group [66]. The latitude and longitude geographic location information of the municipal government was from Tencent Maps (https://lbs.qq.com/getPoint/ (accessed on 26 April 2022)). In order to eliminate the influence of price factors and make the data comparable, all variables in the nominal monetary value such as income and travel cost were converted to actual value using the Consumer Price Index (with 2002 as the base period) in the subsequent empirical analysis.

## 4. Empirical Analysis

### 4.1. Benchmark Regression Results

The results of the benchmark regression are listed in Table 2. Column (1) shows the regression results without adding control variables, and Columns (2) and (3) show the regression results of adding household characteristics and urban characteristics, respectively, as well as other variables. Column (4) shows the regression results with all control variables added. The coefficient of the effect of city size (size) on the cost of household travel is significantly positive in all regressions. The city size index increased by one standard deviation, and the cost of family travel increased by 15.17%. This result is consistent with Hypothesis 1 that city size leads to an increase in the cost of household travel. We will discuss the specific impact mechanism in the next section.

For the other control variables, the average age of household members (age) is negatively correlated with the cost of household travel. This indicates that the ability to travel and willingness to travel decline with age. This results in a decrease in activity participation and travel distance [67], thereby reducing the cost of household travel. The average education level of households (education) is positively correlated with the travel cost. This is because the fact that household members with higher education are willing to increase travel distance for more professional jobs [50,51] leads to an increase in the cost of household travel. The presence of minors in the household (minors) has a significant positive impact on travel costs. The main reason is that households with minors will increase the frequency of travel and use cars to travel more frequently [23,68]. The regression coefficient of family size (family size) is positive and statistically significant. This means that the larger the family size, the higher the travel cost. The regression coefficient of household members in the state-owned economic sector (sector) is positive and statistically significant. Most employees in the state-owned economic sector in China work eight hours a day and five days a week. Therefore, there is time for leisure and entertainment activities, which increases the cost of household travel. Income is an important factor affecting travel [51]. A series of income variables are significantly positively correlated with the cost of household travel. However, there is little difference in the impacts on travel costs.

The regression coefficient of the price level (price) is positive and significant at the 1% level. In areas with high prices, the fares for buses and taxis are relatively high, which has an impact on the cost of household travel. The regression coefficient of government intervention (government) is negative and significant at 5%. This indicates that the government has improved the mixed use of land through urban spatial planning, thereby reducing the cost of household travel [69]. The regression coefficient of the subways (subways) in a city is negative and significant at 5%. As a means of travel, the subway is faster and more convenient than other means of transportation, and the cost is lower. Therefore, the more subways a city has, the lower the cost of household travel. Temperature (temperature) has a positive impact on the cost of household travel, and it is significant at 5%. The possible reason is that the outdoor leisure activities and travel distance of urban residents will increase as the temperature rises [70,71,72]. In addition, as the temperature rises, the use of private cars and public transportation vehicles will increase [68]. As a result, it leads to an increase in the cost of household travel. With the increase in precipitation, urban residents reduce unnecessary travel [70,73,74], thereby reducing the cost of household travel. Therefore, there is a significant negative correlation between precipitation and cost of household travel.

### 4.2. Robustness Testing

In order to more accurately assess the impact of city size on the cost of household travel, we conducted the following robustness test. First, we replaced the explanatory variables and recalculated the cost of household travel (travel cost2). travel cost2 only includes public transportation fees, taxi fees and transportation fuel costs. Column (1) of Table 3 is the regression results of replacing the explained variables. The results show that the impact of city size on the cost of household travel was significantly positive, consistent with the previous benchmark regression results. This indicates that the regression results of this paper are robust.

Second, we made substitutions for the core explanatory variables. We replaced the composite city size index with the size of the urban population (lnpop) and urban build-up area (lnarea). Columns (2) and (3) of Table 3 are the regression results for replacing the core explanatory variables. The results show that the impact of city size on the cost of household travel was significantly positive, consistent with the previous benchmark regression results. This indicates that the regression results of this paper are robust.

Finally, we excluded municipalities, sub-provincial and provincial capital cities, port cities and regions rich in oil and gas resources. Due to the light or gas-burning interference in these cities and regions, the night light data may overestimate the scale of urban space. Therefore, we excluded the aforementioned regions. Column (4) of Table 3 shows the results of excluding municipalities, sub-provincial and provincial capital cities, port cities and regions rich in oil and gas resources. The results show that the impact of city size on the cost of household travel was significantly positive, consistent with the previous benchmark regression results. This indicates that the regression results of this paper are robust.

We must consider the endogeneity problem. First, there is a reverse causality between the city size and the cost of household travel. Households are willing to pay greater travel costs in exchange for a better living environment. The cost of household travel may affect the size of urban build-up area because of the better living environments for residential areas are on the urban fringes. Second, city size and the cost of household travel are simultaneously affected by unobservable factors in the city, so there are endogeneity problems caused by missing variables that affect the estimation results. For example, urban spatial management affects both city size and the cost of household travel, and thus, missing variables will lead to biased model estimates.

The instrumental variables are selected as the time the city has had a railway (IV1) and the distance from each prefecture-level city to the provincial capital (IV2). The reasons for using these two instrumental variables are: First, the railways in cities can greatly promote the agglomeration of population and economic activities and increase the sizes of cities. Therefore, the longer a city has a railway, the larger the scale of the city in the contemporary era. However, railways are links between cities, and they do not directly affect the cost of household travel in the cities. Second, the provincial capital plays a leading role in the economic development of other cities in the province, which in turn affects the land use scale of other cities. This effect weakens as the distance between a provincial capital and other cities increases. However, the distance from other cities to the provincial capital does not affect the cost of household travel within the city. Therefore, it is reasonable to select the time the city has had a railway and the distance from the provincial capital to other cities in the province as the instrumental variables for city size.

The basic principle of the instrumental variable is to use the endogenous explanatory variable (size) to regress the instrumental variables (IV) and the control variables in the first stage to obtain the fitted value (size’); in the second stage, the fitted value (size’) of the first-stage regression and the control variable are regressed by the explanatory variables. Columns (1) and (3) of Table 4 are the first-stage estimation results for IV1 and IV2. The results show that the coefficients of the instrumental variables are significant at 1%. This indicates that there are high correlations between city size and the instrumental variables. In addition, the F values in the first stage are all greater than the critical value 16. Therefore, there is no need to worry about the problem of weak instrumental variables. Columns (2) and (4) of Table 4 are the second-stage regression results for IV1 and IV2. The results show that the impact of city size on the cost of household travel was significantly positive, consistent with the previous benchmark regression results. This indicates that the regression results of this paper are robust.

### 4.3. Mechanism Analysis

#### 4.3.1. The Impact of City Size on the Cost of Household Travel

The existing empirical results showed that city size has increased the cost of resident travel. What is the mechanism that affects the cost of resident travel? In order to further reveal the internal mechanism of the impact of city size on the cost of household travel, a mediating effect model was constructed to test Hypothesis 2 and Hypothesis 3 based on the theoretical analysis and model (1). More details of models (3) and (4) are below:

(3) $$M=\beta_{0}+\beta_{1}{\mathrm{size}}_{\mathrm{jt}}+\beta_{2}\mathrm{CV}+\beta_{3}D+y_{t}+\varepsilon$$

(4) $$\ln\;\left({\mathrm{travel}\;\cos t}_{\mathrm{ijt}}\right)=\gamma_{0}+\gamma_{1}{\mathrm{size}}_{\mathrm{jt}}+\gamma_{2}M+\gamma_{3}\mathrm{CV}+\gamma_{4}D+y_{t}+\varepsilon$$

where M represents the intermediary mechanism variable. It specifically includes spatial distance and congestion. The coefficient $\beta_{1}$ of ${\mathrm{size}}_{\mathrm{jt}}$ in model (3) represents the effect of city size on the intermediary mechanism variables. The coefficient $\beta_{1}$ of ${\mathrm{size}}_{\mathrm{jt}}$ in model (3) and the coefficient $\gamma_{2}$ of the intermediary mechanism variable M in model (4) are both significant. Therefore, the intermediary mechanism variable is the transmission path through which city size increases the cost of household travel. The remaining variables are consistent with the previous definitions.

The coefficient of distance (distance) in Column (1) of Table 5 is significantly positive, indicating that the city size has increased the distance. The coefficients of city size and distance in Column (2) are both positive and statistically significant. The results show that the mediating effect of distance is significant: distance is a mediating variable through which city size affects the cost of household travel. Therefore, we verified Hypothesis 2. The coefficient of congestion (congestion) in Column (3) of Table 5 is significantly positive, indicating that the city size has increased the congestion. The coefficients of city size and congestion in Column (4) are both positive and statistically significant. The results show that the mediating effect of congestion is significant; congestion is a mediating variable through which city size affects the cost of household travel. Therefore, we verified Hypothesis 3.

#### 4.3.2. Reducing the Impact of City Size the Cost of Household Travel

The existing studies have confirmed that the cost of household travel increased with the city size, but it is not clear how to reduce the impact of city size on the cost of household travel. According to the theoretical analysis, a moderating effect model was constructed to test Hypothesis 4 and Hypothesis 5 on the basis of model (1). In particular, we added the interaction term of per capita area of paved roads and city size (size × road) to test Hypothesis 4; see model 5. In order to test Hypothesis 5, we added the interaction terms of the number of public transportation vehicles per 10,000 people and city size (size × bus); see model 6:

(5) $$\ln\;\left({\mathrm{travel}\;\cos t}_{\mathrm{ijt}}\right)=\gamma_{0}+\gamma_{1}{\mathrm{size}}_{\mathrm{jt}}+\gamma_{2}{\mathrm{size}}_{\mathrm{jt}}\times \mathrm{road}+\gamma_{3}\mathrm{CV}+\gamma_{4}D+y_{t}+\varepsilon$$

(6) $$\ln\;\left({\mathrm{travel}\;\cos t}_{\mathrm{ijt}}\right)=\gamma_{0}+\gamma_{1}{\mathrm{size}}_{\mathrm{jt}}+\gamma_{2}{\mathrm{size}}_{\mathrm{jt}}\times \;\mathrm{bus}\;+\gamma_{3}\mathrm{CV}+\gamma_{4}D+y_{t}+\varepsilon$$

Columns (1) and (3) in Table 6 are the OLS estimation results without introducing interactive terms, while Columns (2) and (4) are the OLS estimation results after introducing interactive terms. In Columns (1) to (4), size is significant at the 1% level, all values having a positive impact on cost of household travel. In Column (2) of Table 6, the explanatory variable city size coefficient was significant at 1%, and the coefficient of the interaction of size × road was significantly negative. This indicates that the regulatory effect of the per capita area of paved roads was significant. The connection distances between various areas within the city were shortened in the process of city size as the per capita area of paved roads increased. The accessibility of each area was improved. Therefore, increasing the per capita area of paved roads will restrain the positive effect of the city size on the cost of household travel. This verifies Hypothesis 4.

In Column (4) of Table 6, the explanatory variable city size coefficient was significant at 1%, and the coefficient of the interaction of size×bus was significantly negative. This indicates that the regulatory effect of the number of public transportation vehicles per 10,000 people was significant. The accessibility of public transportation vehicles in areas within the city improved as the number of public transportation vehicles per 10,000 persons increased in the process of city size. This produced the substitution effect of bus travel on private car travel. Urban residents choose public transportation to travel. Therefore, increasing the number of public transportation vehicles per 10,000 people will restrain the positive effect of city size on the cost of household travel. This verifies Hypothesis 5. From the results, the restraining effect of public transportation is stronger than that of per capita road area.

### 4.4. Further Analysis

#### 4.4.1. Heterogeneity Analysis

This paper will further examine the impact of city size on the cost of household travel under different city types. This paper divided cities into sub-provincial (included municipalities and sub-provincial and provincial capital cities) and ordinary prefecture-level cities according to their administrative levels. Based on the above classification, this paper performed a new regression, and the results are shown in Columns (1) and (2) of Table 7. The impact of city size on the cost of household travel for sub-provincial cities is smaller than that for ordinary cities. In fact, the size of sub-provincial cities is larger than that of ordinary prefecture-level cities. The reason is that compared with ordinary prefecture-level cities, sub-provincial cities can obtain more financial funds from their superiors to invest in urban infrastructure construction and improvements. Therefore, the travel cost in sub-provincial cities is lower than that in ordinary prefecture-level cities.

#### 4.4.2. Nonlinear Analysis

We examined the effect of city size on the cost of household travel by grouping. The results show that the impact of city size for sub-provincial cities on the cost of household travel is smaller than that of ordinary prefecture-level cities. Then, we examined whether there is a nonlinear relationship between city size and the cost of household travel. The regression results without adding the control variables are shown in Column (1) of Table 8. Column (2) is the regression results with all control variables added. In Column (2), the results show that both size and size^2^ are significant and are inverted U-shaped. The results further show that with the expansion of city size, the government increased financial investment in infrastructure, improved urban public transport and increased urban road density, which contributed to the formation of scale effects of transportation infrastructure. As a result, the cost of household travel decreases as cities grow in size.

## 5. Conclusions

With the advancement of urbanization and the continuous expansion of city size, the increase in travel costs has increasingly affected the development potential of cities in China. To promote the sustainable development of cities and improve the welfare of residents, this paper selected 153 cities, used the data from the National Bureau of Statistics of China’s urban household surveys from 2002 to 2014, took the household as the unit of analysis and constructed a comprehensive index of city size to explore how the expansion of city size affected the cost of household travel in China. The main findings are summarized below.

First, the results show that city size has increased the cost of household travel, and this paper found that city size affects the cost of household travel in many ways. One reason is that the expansion of city size increases the spatial distance between residences and destinations, which affects the cost of household travel. Another reason is that the congestion has increased with the expansion of the city size, leading to an increase in the cost of household travel. Second, increasing the number of public transportation vehicles and the per capita area of paved roads can restrain the positive effect of city size on the cost of household travel, and the restraining effect of public transport is stronger than that of per capita road area. Third, the impact of city size on the cost of household travel is heterogeneous among different cities. For sub-provincial cities, the impact of city size on the cost of household travel is smaller than that for ordinary prefecture-level cities. In addition, the relationship between city size and the cost of household travel is inverted U-shaped.

The recommended policies are as follows. First, the government should increase the mileage and density of urban roads. In terms of road supply, it is necessary to build fast and large-capacity arterial traffic, as well as branch roads connecting arterial lines and communities, so as to increase the density of urban roads. This can shorten travel distances and reduce travel costs. The second is to develop large-capacity public transportation. Public transportation has the advantages of large passenger capacity and small footprint. According to the spatial distribution of urban residents’ travel needs, reasonably increasing urban public transport vehicles and bus stops, and thereby improving the accessibility of public transport between districts within the city, can reduce personal transport travel. The third is to limit the use of private cars. For cities with severe traffic congestion, the government should control the total number of private cars and impose travel restrictions on private cars. This can improve the efficiency of urban roads and reduce travel costs due to traffic congestion. The fourth is to strengthen urban space management and improve the degree of land use mixing on the street, compressing the distance between residence, employment and commerce. This helps reduce unnecessary travel or minimize the distance travelled. The last is road congestion charges. Vehicles on the road during congested or peak hours will be charged an additional toll. Its purpose is to use the price mechanism to guide traffic demand, restrain the generation of traffic travel and relieve traffic congestion.

There are some limitations in this paper. First, the expansion of the city size increases the travel distance of residents. However, the UHS does not record detailed data on daily travel distances of household members. Therefore, the travel distance of residents is replaced by the average of the linear distances between all grids in the city. Second, the UHS does not contain neighborhood-level traffic facility data. Additionally, most studies using national-level survey data do not include neighborhood-level data [49,75]. Therefore, we did not use variables of neighborhood-level transport facilities in the regression model in this paper. Finally, this paper uses household survey data from 2002 to 2014, which is relatively old. However, this is a common problem faced by all researchers using microdata in their research. In recent years, increasing amounts of big data have been applied to the research on resident travel [76,77,78]. More detailed travel information on residents can be recorded in big data. Big data rarely directly include socioeconomic attributes, but the survey’s data provide a wealth of information on individual socioeconomic characteristics. Therefore, the combination of survey data and big data can better evaluate the impacts of city size, urban spatial structure and urban built environment on resident travel. This is the focus of future research. With the COVID-19 pandemic, many companies have turned to telecommuting. This can alleviate the cost of commuting due to job-housing separation [79]. However, there is no clear answer to the impact of online telecommuting on other travel [79,80], which may be another focus of future research.

## Figures and Tables

**Table 1:** Descriptive statistics.

Variable	Mean	Std. Dev.	Min	Max
ln (travel cost)	5.326	2.245	0.000	13.044
size	1.551	2.121	0.003	10
age	41.456	13.298	7.600	90
education	5.013	1.212	0.500	9
minors	0.457	0.498	0	1
family size	2.903	0.856	1.000	12.000
sector	0.542	0.498	0	1
wage	8.399	3.751	0	13.178
operational	0.916	2.843	0	13.304
property	1.318	2.875	0	14.390
transfer	7.324	2.675	0	13.221
density	7.563	0.878	4.007	9.470
price	1.551	0.473	0.811	2.890
government	0.137	0.298	0.015	8.911
PM 2.5	3.741	0.445	1.896	4.531
subway	0.352	0.721	0	2.773
spatial form	0.735	0.165	0.167	1
river	0.249	0.432	0	1
terrain	0.500	0.676	0.001	3.814
temperature	2.641	0.324	1.443	3.163
precipitation	9.043	0.429	7.554	10.091
latitude	34.359	6.350	21.270	47.728
longitude	116.206	6.551	98.290	131.141
disbeijing	6.266	1.899	0	7.740
disshanghai	6.535	1.443	0	7.740
congestion	3.064	1.371	0.182	5.752
distance	2.492	0.776	0.088	4.073
road	2.278	0.506	0.642	4.302
bus	2.159	0.659	0.278	4.714

**Table 2:** The benchmark estimation results.

	(1)	(2)	(3)	(4)
Variables	ln (Travel Cost)	ln (Travel Cost)	ln (Travel Cost)	ln (Travel Cost)
size	0.2332 ***	0.1566 ***	0.2160 ***	0.1517 ***
	(0.0262)	(0.0237)	(0.0313)	(0.0297)
age		−0.0129 ***		−0.0144 ***
		(0.0010)		(0.0010)
education		0.3313 ***		0.3596 ***
		(0.0080)		(0.0080)
minors		0.2669 ***		0.2951 ***
		(0.0212)		(0.0216)
family size		0.0704 ***		0.0507 ***
		(0.0113)		(0.0111)
sector		0.1907 ***		0.2330 ***
		(0.0208)		(0.0204)
wage		0.0743 ***		0.0774 ***
		(0.0034)		(0.0034)
operational		0.0522 ***		0.0538 ***
		(0.0031)		(0.0032)
property		0.0512 ***		0.0706 ***
		(0.0039)		(0.0033)
transfer		0.0924 ***		0.1049 ***
		(0.0047)		(0.0049)
density			0.0179	0.0150
			(0.0256)	(0.0235)
price			0.6356 ***	0.6897 ***
			(0.1974)	(0.1770)
government			−0.0946 ***	−0.0669 **
			(0.0350)	(0.0299)
PM 2.5			−0.0265	−0.1120
			(0.1174)	(0.1060)
subway			−0.1099	−0.1342 **
			(0.0755)	(0.0671)
spatial form			−0.2218	−0.1018
			(0.1392)	(0.1176)
river			0.0949 *	0.0733
			(0.0548)	(0.0485)
terrain			−0.0124	−0.0260
			(0.0774)	(0.0737)
temperature			0.6965 *	0.7915 **
			(0.4064)	(0.3711)
precipitation			−0.6754 ***	−0.6031 ***
			(0.1459)	(0.1324)
latitude			−0.0498	−0.0319
			(0.0350)	(0.0321)
longitude			0.0140	0.0248 *
			(0.0157)	(0.0140)
disbeijing			0.0480	0.0370
			(0.1640)	(0.1499)
disshanghai			0.0014	−0.0671
			(0.1127)	(0.1069)
Constant	4.5344 ***	1.2714 ***	8.6278 **	3.4744
	(0.1646)	(0.1634)	(3.4657)	(3.2234)
Observations	100,869	100,869	100,869	100,869
R-squared	0.1134	0.2483	0.1178	0.2269
province FE	Yes	Yes	Yes	Yes
Year FE	Yes	Yes	Yes	Yes

Robust standard errors in parentheses *** *p* < 0.01, ** *p* < 0.05, * *p* < 0.1.

**Table 3:** The robustness test results.

	(1)	(2)	(3)	(4)
Variables	ln (Travel Cost2)	ln (Travel Cost)	ln (Travel Cost)	ln (Travel Cost)
size	0.2173 ***			0.5351 ***
	(0.0374)			(0.0832)
lnarea		0.1167 ***		
		(0.0286)		
lnpop			0.1268 ***	
			(0.0308)	
Constant	−2.1333	2.3398	2.0618	−8.7187 *
	(3.8525)	(3.1787)	(3.2002)	(5.1021)
Observations	100,869	100,869	100,869	50,564
R-squared	0.2414	0.2272	0.2265	0.2174
Control variables	Yes	Yes	Yes	Yes
province FE	Yes	Yes	Yes	Yes
Year FE	Yes	Yes	Yes	Yes

Robust standard errors in parentheses *** *p* < 0.01, * *p* < 0.1. China’s land oil wells are concentrated in Tianjin, Heilongjiang, Shandong, Shaanxi and Xinjiang.

**Table 4:** The endogeneity test results.

	(1)	(2)	(3)	(4)
Variables	Size	ln (Travel Cost)	Size	ln (Travel Cost)
IV1	0.1458 ***			
	(0.0124)			
IV2			−0.2053 ***	
			(0.0140)	
size		0.3388 ***		0.1631 ***
		(0.1032)		(0.0538)
Constant	3.8147	2.7608	6.3539 **	3.4308
	(3.0929)	(3.2149)	(2.6947)	(3.2289)
Observations	100,869	100,869	100,869	100,869
R-squared	0.9327	0.2246	0.9497	0.2269
Control variables	Yes	Yes	Yes	Yes
province FE	Yes	Yes	Yes	Yes
Year FE	Yes	Yes	Yes	Yes
F value	137.68 ***		214.06 ***	

Robust standard errors in parentheses *** *p* < 0.01, ** *p* < 0.05.

**Table 5:** The regression results for the mediation effects.

	(1)	(2)	(3)	(4)
Variables	Distance	ln (Travel Cost)	Congestion	ln (Travel Cost)
size	0.3769 ***	0.0745 **	0.5999 ***	0.1018 ***
	(0.0387)	(0.0292)	(0.0620)	(0.0343)
distance		0.2049 ***		
		(0.0449)		
congestion				0.0832 **
				(0.0398)
Constant	8.9240 ***	1.6461	11.9890 ***	2.4771
	(2.8901)	(3.0981)	(3.4877)	(3.2143)
Observations	100,869	100,869	100,869	100,869
R-squared	0.7668	0.2280	0.8954	0.2271
Control variables	Yes	Yes	Yes	Yes
province FE	Yes	Yes	Yes	Yes
Year FE	Yes	Yes	Yes	Yes

Robust standard errors in parentheses *** *p* < 0.01, ** *p* < 0.05.

**Table 6:** The regression results for the moderating effects.

	(1)	(2)	(3)	(4)
Variables	ln (Travel Cost)	ln (Travel Cost)	ln (Travel Cost)	ln (Travel Cost)
size	0.1549 ***	0.3563 ***	0.1173 ***	0.5064 ***
	(0.0291)	(0.0576)	(0.0293)	(0.0609)
size × road		−0.0841 ***		
		(0.0196)		
size × bus				−0.1605 ***
				(0.0216)
road	0.1688 ***	0.2825 ***		
	(0.0502)	(0.0593)		
bus			0.1833 ***	0.3494 ***
			(0.0370)	(0.0443)
Constant	2.0729	1.5602	1.8637	−0.5581
	(3.1408)	(3.0808)	(3.0499)	(2.9566)
Observations	100,869	100,869	100,869	100,869
R-squared	0.2539	0.2545	0.2542	0.2564
Control variables	Yes	Yes	Yes	Yes
province FE	Yes	Yes	Yes	Yes
Year FE	Yes	Yes	Yes	Yes

Robust standard errors in parentheses *** *p* < 0.01.

**Table 7:** The heterogeneous regression results.

	Sub-Provincial Cities	Ordinary Cities
Variables	ln (Travel Cost)	ln (Travel Cost)
size	0.0823 *	0.5299 ***
	(0.0471)	(0.0674)
Constant	40.0663 ***	−6.2588 **
	(10.8097)	(3.1745)
Observations	37,940	62,929
R-squared	0.2523	0.2015
Control variables	Yes	Yes
province FE	Yes	Yes
Year FE	Yes	Yes

Robust standard errors in parentheses *** *p* < 0.01, ** *p* < 0.05, * *p* < 0.1.

**Table 8:** The nonlinear regression results.

	(1)	(2)
Variables	ln (Travel Cost)	ln (Travel Cost)
size	0.3557 ***	0.2374 ***
	(0.0367)	(0.0446)
size^2^	−0.0234 ***	−0.0136 ***
	(0.0047)	(0.0047)
Constant	4.5938 ***	2.9307
	(0.1556)	(3.1868)
Observations	100,869	100,869
R-squared	0.1149	0.2273
Control variables	No	Yes
province FE	Yes	Yes
Year FE	Yes	Yes

Robust standard errors in parentheses *** *p* < 0.01.

## Data Availability

The datasets used and analyses during the current study are available from the corresponding author on reasonable request.
